# Predicting deleterious missense genetic variants via integrative supervised nonnegative matrix tri-factorization

**DOI:** 10.1038/s41598-021-03230-x

**Published:** 2021-12-09

**Authors:** Asieh Amousoltani Arani, Mohammadreza Sehhati, Mohammad Amin Tabatabaiefar

**Affiliations:** 1 Department of Bioelectric and Biomedical Engineering, School of Advanced Technologies in Medicine, Isfahan University of Medical Sciences, Isfahan, Iran; 2 Student Research Committee, School of Advanced Technologies in Medicine, Isfahan University of Medical Sciences, Isfahan, Iran; 3 Department of Bioinformatics, School of Advanced Technologies in Medicine, Isfahan University of Medical Sciences, Isfahan, Iran; 4 Deputy of Research and Technology, GTaC Corp, Isfahan University of Medical Sciences, Isfahan, Iran; 5 Department of Genetics and Molecular Biology, School of Medicine, Isfahan University of Medical Sciences, Isfahan, Iran

**Keywords:** Computational biology and bioinformatics, Systems biology

## Abstract

Among an assortment of genetic variations, Missense are major ones which a small subset of them may led to the upset of the protein function and ultimately end in human diseases. Various machine learning methods were declared to differentiate deleterious and benign missense variants by means of a large number of features, including structure, sequence, interaction networks, gene disease associations as well as phenotypes. However, development of a reliable and accurate algorithm for merging heterogeneous information is highly needed as it could be captured all information of complex interactions on network that genes participate in. In this study we proposed a new method based on the non-negative matrix tri-factorization clustering method. We outlined two versions of the proposed method: two-source and three-source algorithms. Two-source algorithm aggregates individual deleteriousness prediction methods and PPI network, and three-source algorithm incorporates gene disease associations into the other sources already mentioned. Four benchmark datasets were employed for internally and externally validation of both algorithms of our predictor. The results at all datasets confirmed that, our method outperforms most state of the art variant prediction tools. Two key features of our variant effect prediction method are worth mentioning. Firstly, despite the fact that the incorporation of gene disease information at three-source algorithm can improve prediction performance by comparison with two-source algorithm, our method did not hinder by type 2 circularity error unlike some recent ensemble-based prediction methods. Type 2 circularity error occurs when the predictor annotates variants on the basis of the genes located on. Secondly, the performance of our predictor is superior over other ensemble-based methods for variants positioned on genes in which we do not have enough information about their pathogenicity.

## Introduction

Next-generation sequencing (NGS) as a cost-effective sequencing technology and straightforward performing has rapidly enhanced the discovering of various types of variants. They include insertions, deletions and single nucleotide variants (SNVs) which most of them stay in exome region^1^. Nonsynonymous single nucleotide variants (nsSNVs) that cause some changes in amino acid sequence of corresponding protein are regarded as missense variants^2^. As a result, interpreting the involvement of nsSNVs in human diseases either Mendelian or complex diseases has the potential to help better models for personalized medicine procedures^3^. Moreover, wet lab experiments for huge amount of variants are time consuming and expensive because they squander many work forces along with reserves.

Variant prioritization tools such as ANNOVAR^4^ and Genome Mining(GEMINI)^5^ are based on popular hard-filtering strategies which select nsSNVs and loss-of-function variants which should not appear in unaffected people^6^. Furthermore, specified mode of inheritance and filtrated rare variants based on the value of allele frequency are considered as other criteria for removal of neutral variants.

Inasmuch as many causal variants remain undetectable, various machine learning methods have been developed^7–17^. The state-of-the-art nsSNV prediction methods, integrate association information between genes harbored the variants and diseases into variant-level information including sequence-based, structure-based and network features or available functional predictors of variants^18–20^. The hypothesis behind these approaches is that variants placed in genes which are related to each other, have alike properties. Thus, the association score could be calculated according to the similarities of corresponding genes to recognized disease genes, which is utilized in gene prioritization methods^21^. Similarity measurement makes use of various data, protein–protein interaction, gene expression, gene involvement in a same pathway, and phenotypes information. The final destination of all variant effect prediction approaches is phenotypical interpretation of the effect of protein perturbation at human being. As a result, Phenotype-driven computational strategies^22–24^ identify gene–disease associations use phenotypes in order to completely show interactions taking place in an organism over multiple levels of organization. On the other hand, all variants in corresponding disease genes are not pathogenic. So, to discriminate pathogenic variant from neutral ones placed in the same gene, variant features level play an important role.

As already discussed, some notable points could be extracted from all kinds of available variant-effect prediction algorithms. Firstly, although recent methods, especially ensemble ones, and the already mentioned group regarding the disease-gene relations, have high performance at benchmark datasets, it has been proved that recent de novo nsSNV variants cannot be certainly recognized. In some cases rare neutral nsSNVs detected as positive cases mistakenly. On the other hand, tools rely on knowledge of gene harbored specific variants are incapable to distinguish variants within the same protein. Therefore, their validity in clinical approaches cannot be guaranteed^25^. Secondly, network features obtained from protein–protein interaction (PPI) are a kind of information which have received less attention, while they play a pivotal role in variant classification, because disturbed protein interactome regularly results in disease. If a variant is related to more proteins at the network, it will be more probable to disturb such function and to be deleterious. The most up-to-date nsSNP predictors are using topological features measuring the importance of proteins in the PPI network like betweenness, closeness, degree and so on^26–29^. But we need to consider the whole PPI network topology to improve the variant effect prediction results. Thirdly, as a result of the essential needs for aggregation of variant level features into preceding information of the disease and gene(s) engaged, a great number of heterogeneous input data sources has produced. Hence, there is no systematic procedure to aggregate such knowledge to simultaneously take into account the formation of all input data sources and shape an accurate workflow of deleterious variant detection^22^.

To resolve the above-described problems, an appropriate machine learning algorithm is indispensable in order to have better assessment of the complex relationship of variant level information, PPI interaction, and disease-gene relations for variant interpretation. We designed a data fusion algorithm which is based on non-negative matrix tri-factorization (NMTF). The NMTF algorithm aims to estimate the input matrix indicating relation of two different kinds of data source by product of three low-dimensional matrices and clusters both sources simultaneously. It can be extended to more than two sources of information. NMTF based methods are usually utilized for co-clustering heterogeneous datasets or to discover potential relations between different kinds of input data in many bioinformatics fields including discovery of biological data in order to find the origin of diseases, drug investigation, and similar^30–33^.

In the case of classification settings, the classical NMF algorithms were applied by merging group labels into the framework^34–36^. In comparison with NMTF, it comes up with two matrices which their products could well approximate the non-negative input data matrix. For integration applications, these type of algorithms intrinsically do not have the capability to be used in heterogeneous data sources. On the other hand, NMTF-based algorithms do not have this limitation for such datasets. Therefore, at our previous work we designed an aggregation workflow of many types of data sources to identify deleterious nsSNPs which we called supervised NMTF, sNMTF^37^. At that work, the NMTF algorithm was used to generate a feature map at a low dimension space on two networks. This forces the algorithm to apply additional classifier in new feature spaces. So, we do not directly take advantage of network information and, In addition a classifier at the workflow of supervised NMTF makes algorithm more complex. Moreover, our sNMTF classifier is unable to properly detect deleterious variants positioned on genes which are not pathogenic because of the lack of information.

In this study, an original supervised non-negative matrix tri-factorization deleterious variant prediction method was recommended, concerning all kinds of information. It takes into account the label information for objective function at learning procedure. Subsequently, at the testing phase of our algorithm, we employed the learned factorized matrices to characterize unknown nsSNP samples. Two versions of algorithm were investigated. At first step, the two-source algorithm, exploited variant level features including available variant effect prediction scores and PPI network to construct score-score as well as variant-variant network. Afterwards, three-source algorithm was shaped by joining gene-disease association to previous two-source algorithm by means of variant-disease relation matrix and disease-disease network to advance the separation of nsSNVs into deleterious and neutral variants.

Current study covers our four benchmark datasets employed for validation of our work. The formation of score, variant and disease networks and origination of function prediction scores, PPI network and disease-gene relations were outlined. Subsequently, we depicted the structure of our two prediction algorithms in details. Afterwards, the obtained prediction resulted from our both constructed classifications is presented, and discussed. We compared two algorithms with each other and existing prediction scores. Finally, we verified the effect of inserting new sources on variant effect prediction. At the end, we searched how algorithms are confronted by circulatory errors through defining two separated test datasets.

## Material and methods

All methods were carried out in accordance with relevant guidelines and regulations. The current section deals with outlining the datasets and describing our proposed sNMTF-VAR scheme. Afterwards, considering the point that our classification methods are on the basis of non-negative matrix tri-factorization, we proceed with a formal representation of derivation for our two algorithms.

### Input data

We performed our models on two sets of datasets which are publicly available and are commonly used as benchmark datasets. The training dataset on which we applied parameter tuning for our algorithms, did our cross-validation on, and testing dataset for external validation. The training dataset consists of 14,894 deleterious nsSNPs as true positive (TP) observations and 23,956 neutral nsSNPs as true negative (TN) ones. The data are the combination of datasets that were used on function prediction methods, MetaSVM^13^ and iFish^38^ which were obtained from Uniprot, HGMD, and dbSNP datasets.

We collected four test datasets to validate our workflow. Testing dataset I, is the data used on evaluation of MetaSVM named as the same, composed of 120 newly Mendelian disease-causing nsSNP reported in Nature Genetics papers, and 118 neutral variants recently discovered from healthy people from Cohorts for Heart and Aging Research in Genomic Epidemiology (CHARGE) sequencing project^39,40^. Testing dataset II was applied on MetaSVM method as additional dataset 1.

To evaluate how our method could be affected by circulatory type2, we benefited two other datasets, testing dataset III and testing dataset IV, applied on iFish paper for this purpose as SwissvarFilteredMix and VaribenchSelectedPure^38^.

All prediction scores needed for each dataset were taken from VarCards database^41^. This integrated online database can straightforwardly regain general genetics together with clinical knowledge for the included variants. The information comprised of gene-disease relation, functional effects of variants, allele frequency and phenotype-related data.

As some values obtained from VarCards have been missed, we discarded them in all datasets. Overlapping variants of Training dataset and all of testing datasets were removed. Since all the test datasets were applied in previous ensemble based publications, we are sure that our Testing datasets and training datasets of function prediction scores which our predictor are composed of, do not have any common samples. Testing dataset I was collected from recently available projects and publications, it is improbable to be exploited for training of prediction procedures which our algorithms implement in. Furthermore, In the case of testing dataset II and testing dataset III, we know that iFish method excluded the common variants of these datasets and the data which Polyphen2 was trained to^42^. Besides, Sift, MutationAssessor, PROVEAN, GERP++, phyloP, phastCons and SiPhy^43–49^ are conservation scores and did not apply any machine learning classifier to train variants. The final statistics of all our datasets is illustrated at Table 1.

**Table 1:** Summary of training and testing data used in the current study.

	Training dataset	Testing dataset I	Testing dataset II	Testing dataset III	Testing dataset IV
Number of neutral variants	23,956	118	15,785	1063	3114
Number of deleterious variants	14,894	120	13,999	1077	2060
Number of variants placed on disease related genes (percentage to all)	35,256 (90%)	197 (82%)	27,307 (91%)	2051 (95%)	4718 (90%)
Number of variants placed on pure genes (percentage to all)	20,555 (53%)	238 (100%)	20,062 (67%)	350 (16%)	5174 (100%)
Total variants	38,850	238	29,784	2140	5174
Number of pure genes (percentage to all)	7404 (83%)	166 (100%)	6678 (93%)	107 (36%)	2497 (100%)
Total genes	8867	166	7166	293	2497
Source	Uniprot, HGMD 2015.3, dbSNP142	Recent Nature Genetics publications, CHARGE database	Varibench	Uniprot	Varibench

### Data matrices

To implement our algorithm, we considered three data sources, PPI, variant effect prediction scores, and diseases. We constructed three intra-type networks, variant-variant (V–V), score-score(S–S), and disease-disease (D–D) network. In addition, we regarded two relation matrices that are inter-type connection between variants-scores (R_VS_) and variants-disease (R_VD_).

### Intra-type connection networks

The variant-variant (V–V) network was made base on PPI network as Leal et al. did at their study^32^. For any pair of variants, the harboured genes were mapped to corresponding proteins on PPI network. If two variants were placed at the same genes, they can get connected to each other. At the other case, if two corresponding proteins were connected to each other at PPI network, variants were linked to each other with the same weight of connection relating two proteins at PPI network. All of this kind of edges were weighted with value of one, then divided by n-1, in which n is the number of variants in the gene. The weighting works toward decreasing bias in the node degree once many variants located on a gene. Our strategy at constructing V-V network is illustrated at Figure S1. PPI interaction information was obtained from STRING database, version 11.0. Our PPI network consists of as many as 1,015,686 interactions between 13,499 proteins with a confidence score higher than 0.15.

In our S–S network, each node is a feature vector that includes functionally damaging scores of all variants. The type of scores we consider are including SIFT, Polyphen2, MutationAssessor, LRT, PROVEAN, GERP +  + , phyloP, phastCons and SiPhy . Furthermore, the dimension of the S_S network was found to be 9*9.

As each of these scores span on different ranges, a linear transformation was applied in these scores to arrange all of scores in the same interval of [0, 1] according to the following formula:

1 $$ A^{^{\prime}} = \frac{{A_{{}} - A_{\min } }}{{A_{\max } - A_{\min } }} $$

In which A is the score value for deleteriousness prediction score. Also, maximum and minimum values of each feature score are defined in respective by $A_{\max }$ and $A_{\min }$. The maximum and minimum values of SIFT and LRT scores imply the functionally damaging strength of a score at the opposite side. Thus, the SIFT and LRT scores were changed into 1-SIFT and 1-LRT prior to linear transformation. At the S_S network, the connection weight between a pair of scores for example SIFT and Polyphen, was measured by Manhattan distance.

For the case of our three-sources algorithm, we constructed disease-disease network, D-D, using the DigGenet database^50^. The weights of edges in this network, were indicated as the association between two diseases. Association score for each disease pair, is evaluated by a Jaccard Index, measuring the fraction of shared, among all diseases, to total variants related to Disease 1 and 2. It has been shown in Eq. (2):

2 $$ Jaccard_{V} = \frac{{V_{1} \cap V_{2} }}{{V_{1} \cup V_{2} }} $$

where V1 and V2 are the associated variants to disease 1 and 2, respectively.

### Interrelated matrices

To constitute relation matrix, R_VS_, we used values of normalized functionally damaging scores, A’. For each variant we have as many as 9 scores which are the nodes of S–S network.

For the three-source algorithm, it was necessary to connect variants and diseases through relation matrix, R_VD_. As a result, we mapped each variant to identified genes which is located in. Also using DisGeNET database (https://www.disgenet.org) associations between these genes and diseases were recognized. We would connect the nodes of two D-D and V-V network, if the corresponding gene of a variant is linked to diseases presented at the gene-diseases DisGeNET database. The construction of R_VD_ is the same with what has been illustrated at the Amousoltani et. al^37^.

### Algorithm 1: supervised matrix factorization with two sources of variants and previous deleterious prediction scores

**Figure 1:**
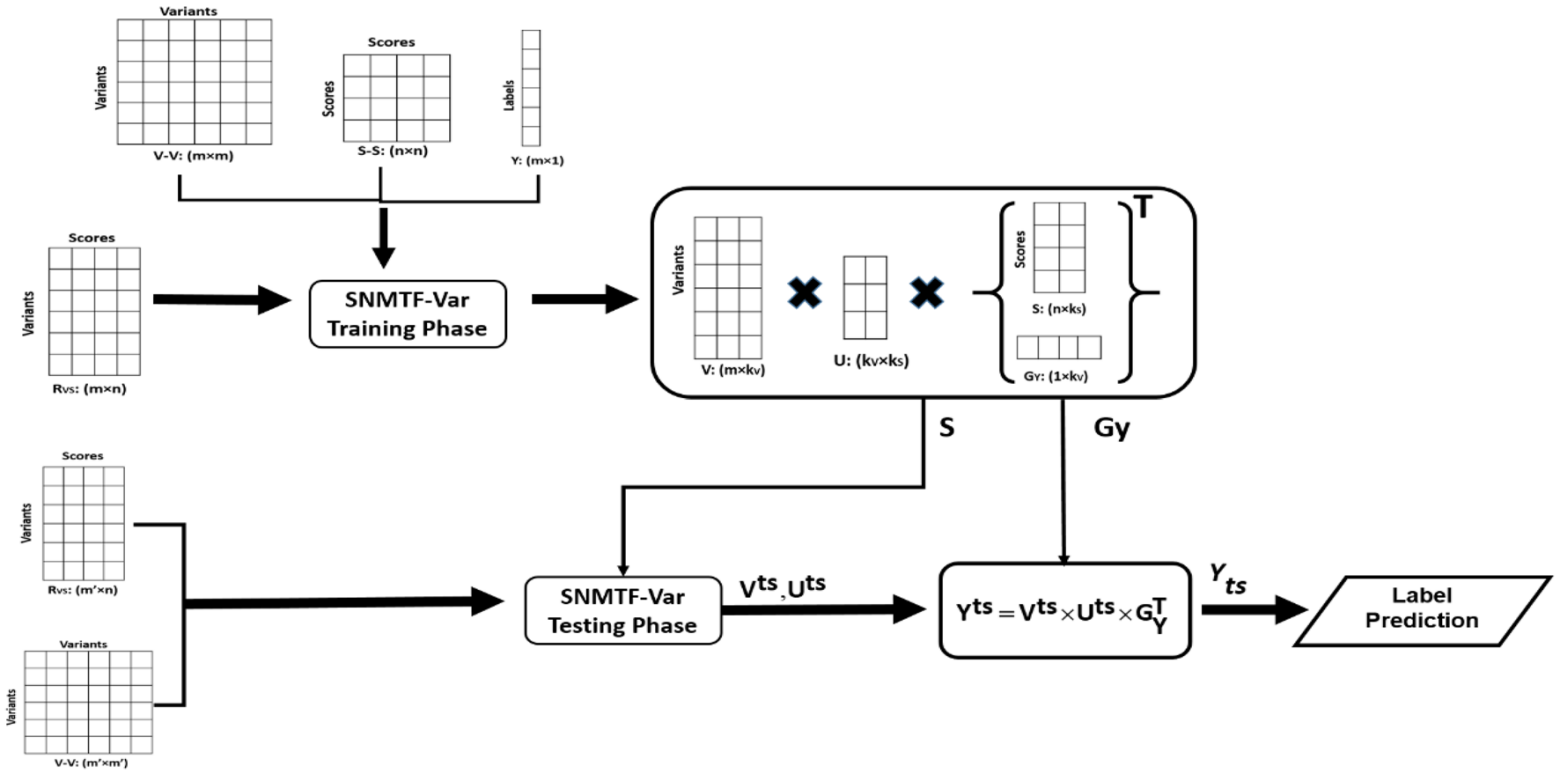
Schematic of two-source algorithm. It shows the training and testing part of the algorithm. *The figure is created using Microsoft PowerPoint* 2013.

Our supervised NMTF based method, Algorithm 1, were consisted of two training and testing phases. We decomposed the relation and label matrices to three nonnegative matrices by nonnegative matrix tri factorization, in the training step. Moreover, the intra type networks (S–S and V–V networks) include in objective function to minimize the error function. Except for class indicator matrix of labels and scores which kept fixed at testing phase, other factorized matrices would be predicted. The second objective function which did not contain labels, at the test phase, only factorized the relation matrix. Finally, with class indicator matrix of variant and label data which were calculated at testing and training phases, respectively, testing variant labels were predicted. The roadmap of our proposed method for two data sources of variant and scores, has been illustrated in Fig. 1. Each step is described in detail in the following.

In the both training and testing phases, we factorized the relation matrix, R_VS_. in the training phase, we also factorized the labels(Y) into three nonnegative matrix factors as given:

3 $$ R_{VS} = VUS^{T} $$

4 $$ Y = VUG_{Y}^{T} $$

At the above Eqs. (3) and (4), the dimension of R_VS_, V, U, S and G_Y_ are m × n, m × k_V_, k_V_ × k_S_, n × k_S_ and 1 × k_S_, respectively.

For two sources of data, variant and score, V, S and G_Y_ are the cluster indicator matrices of the first, second dataset and labels, respectively. U is a compressed version of the both relation matrix (R_VS_) and labels(Y) that implies interactions between a stated variant modules (cluster) in respect of a score cluster. The amount of score and variant clusters comes up with rank parameters k_s_ together with k_V_, respectively.

Our aim is to reduce the difference of approximation and original relation matrices. To substantially improve the learning functioning, In addition to intertype variant-score relation matrix, we incorporated our two intra-types connection data, namely variant-variant (V) and score-score(S) networks as constraint matrices into the objective function. A constraint matrix explains association between objects of the identical type. These two matrices which were represented in the form of Laplacian matrices of V and S networks, L_V_ and L_S,_ respectively, were used for regularization and not decomposed. Nevertheless, the constraint terms ensure that the representation matrices of interacted scores and variants are in the vicinity of each other in the presented Euclidean space.

Given a relation matrix R_VS_, label vector and two constraint matrices, algorithm simultaneously factorizes relation matrix and label vector. This could be accomplished by solving the proposed objective function:

5 $$ J_{tr} = \begin{array}{*{20}c} {\mathop {\min }\limits_{{V,S,G_{Y} \ge 0}} } & {\left\| {R_{VS}^{tr} - V^{tr} U^{tr} S^{T} } \right\|} \\ \end{array}_{F}^{2} + \left\| {Y_{{}}^{tr} - V^{tr} U^{tr} G_{Y}^{T} } \right\|_{F}^{2} + \gamma_{1} tr\left( {V^{tr} L{}_{V}^{tr} V^{T(tr)} } \right) + \gamma_{2} tr\left( {SL_{S}^{tr} S^{T} } \right) $$

In which || · ||, tr(·) stand for the Frobenius norm and trace, respectively. $\gamma_{1}$ and $\gamma_{2}$ are regularization parameters identify the quantity of influence for each V-V and S–S networks.

The computed low-dimension matrices at the training phase were reutilized for prediction of test samples in our algorithm. While we computed the S and GY matrices based on algorithm 1 using the training data, we estimated the deleteriousness of the unseen test variants by *Y*^*ts*^. Making use of the test data sources, the relation matrix, ${\mathrm{R}}_{\mathrm{VS}}^{\mathrm{ts}} ,$ and V-V network, the test variants were mapped into the learned low dimensional space spanned by V matrix. So the objective function at Eq. (5) for the test step will reduce to the:

6 $$ J_{ts} = \begin{array}{*{20}c} {\mathop {\min }\limits_{V \ge 0} } & {\left\| {R_{VS}^{ts} - V^{ts} US^{T} } \right\|} \\ \end{array}_{F}^{2} + \gamma_{1} tr\left( {V^{ts} L_{V}^{ts} V^{(ts)T} } \right) $$

Solving this optimization function, we gained the V^ts^ and U^ts^ matrices from equations S6, S7 to predict test labels using Eq. (7).

7 $$ Y^{ts} = V^{ts} \times U^{ts} \times G_{Y}^{T} $$

### Algorithm 2: supervised matrix factorization with three sources of variants, previous deleterious prediction scores and diseases

**Figure 2:**
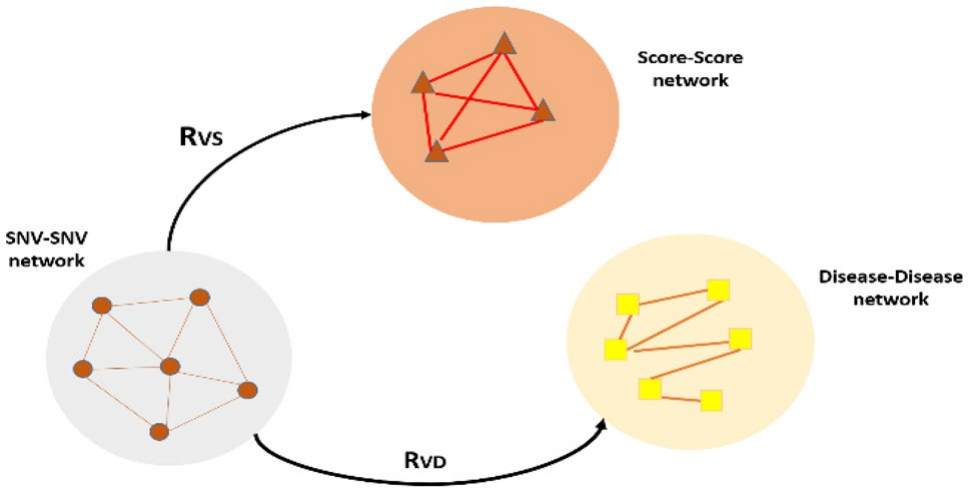
Configuration of our method for three types of data sources. R_VD_ associates SNV-SNV and Disease-Disease networks. R_VS_ shows the relation matrix which connects SNV-SNV and Score-Score networks. *The figure is created using Microsoft PowerPoint* 2013.

At the next stage, for our second algorithm we added other database to our two networks, disease information. So we had three intra related matrices, variant-variant, score-score, and disease-disease networks. We connected these three networks via two relation matrices, R_VS_ and R_VD_. The schematic of networks that include in algorithm 2 is shown at Fig. 2.

As the disease network was added to our algorithm 1, the objective function will change from Eq. (5) to:

8 $$ J_{tr} = \mathop {\min }\limits_{V,S \ge 0} \left\| {R_{VS} - VU_{1} S^{T} } \right\|_{F}^{2} + \left\| {R_{VD} - VU_{2} D^{T} } \right\|_{F}^{2} + \left\| {Y - VU_{1} G_{Y}^{T} } \right\|_{F}^{2} + \gamma_{1} tr\left( {VL_{V} V^{T} } \right) + \gamma_{2} tr\left( {SL_{S} S^{T} } \right) + \gamma_{3} tr\left( {DL_{D} D^{T} } \right) $$

In which $\gamma_{3}$ clarifies the extent of influence and L_D_ is the laplacian matrix of D network. Similar to the algorithm 1, at the training stage, we decompsed the ralation matrices, ${\mathrm{R}}_{\mathrm{VS}}$ and ${\mathrm{R}}_{\mathrm{VD}}$ to V, U_1_, U_2_ and D. Also, the label matrix was factorized to V,U_1_ and G_Y_.

When we derive the desired matrices from training phase, we use learning matrices, $\mathrm{S},\mathrm{D and }{\mathrm{G}}_{\mathrm{Y}}$ to predict test variant labels. As the, $\mathrm{S},\mathrm{D and }{\mathrm{G}}_{\mathrm{Y}}$ are fixed, the test objective function will be as following:

9 $$ J_{ts} = \begin{array}{*{20}c} {\mathop {\min }\limits_{V \ge 0} } & {\left\| {R_{VS}^{ts} - V^{ts} U_{1}^{ts} S^{T} } \right\|} \\ \end{array}_{F}^{2} + \left\| {R_{VD}^{ts} - V^{ts} U_{2}^{ts} D^{T} } \right\|_{F}^{2} + \gamma_{1} tr\left( {V^{ts} L{}_{V}^{ts} V^{(ts)T} } \right) $$

So, the predicted labels was determined by:

$$ Y^{ts} = V^{ts} \times U_{1}^{ts} \times G_{Y}^{T} $$

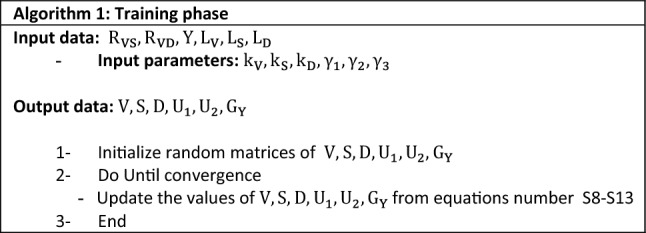



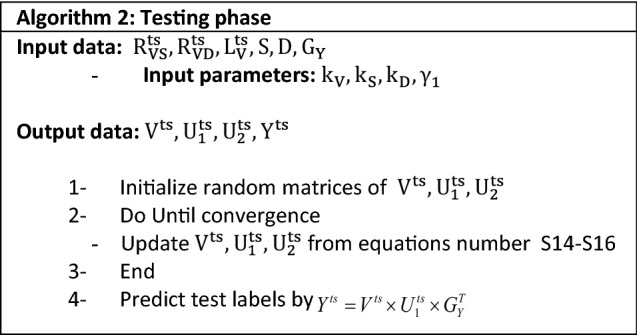


### Experimental settings

As described at the parameter selection and penalization parameters sections in the supplementary material, regularization parameters and factorization ranks were opted in a grid search at training dataset. Moreover, we initialized the random matrices at both algorithms of our approach by the random A-col method.

Our method not only was internally tenfold cross-validated on train dataset but also was compared its both algorithms performance with each other and other deleterious prediction methods in independent external validation data. At the cross validation, we followed the strategy that one part of all genes contain the available variants, was hold for testing data as unseen data, and other nine parts as training dataset. Thus, to counteract the consequence of information leakage between train and test data, we choose all variants located at a given gene for training and testing parts of each datasets.

We evaluated the outcomes of prediction via measures including area under curve (AUC), accuracy (ACC), specificity or true negative rate (TNR), sensitivity or true positive rate (TPR), false positive rate (FPR), false negative rate (FNR), Matthews Correlation Coefficient (MCC) as well as F1 score. MCC takes measurement of the correlation of real and predicted label in classification which whose value is between − 1 and 1, where  − 1 stands for complete difference of prediction and observation, and 1 denotes for perfect prediction.

$$ \begin{gathered} Accuracy = \frac{TP + TN}{{TP + TN + FN + FP}} \hfill \\ precision = \frac{TP}{{TP + FP}} \hfill \\ sensitivity/TPR = \frac{TP}{{TP + FN}} \hfill \\ specificity/TNR = \frac{TN}{{FP + TN}} \hfill \\ F1 - score = \frac{2TP}{{2TP + FP + FN}} \hfill \\ MCC = \frac{TP \times TN - FP \times FN}{{\sqrt {(TP + FP)(TP + FN)(TN + FP)(TN + FN)} }} \hfill \\ \end{gathered} $$

## Results

### Survey on effects of variant level and gene-disease association information on proposed method

To investigate the performance of both two-source and three-source of our proposed method, we formed three testing datasets making use of various sources in order to evaluate generalizability of our proposed method. The resulted measures are illustrated at Table 2.

**Table 2:** The Performance of our method in both two-source and three-source algorithms.

	Three-source algorithm					Two-source algorithm				
	Training data	Testing data 1	Testing data 2	Testing data 3	Testing data 4	Training data	Testing data 1	Testing data 2	Testing data 3	Testing data 4
ACC	0.84 ± 0.01	0.86	0.91	0.73	0.74	0.79 ± 0.01	0.84	0.83	0.72	0.70
Precision	0.76 ± 0.02	0.82	0.92	0.72	0.76	0.72 ± 0.02	0.77	0.83	0.70	0.66
Sensitivity	0.78 ± 0.01	0.92	0.87	0.73	0.46	0.72 ± 0.01	0.95	0.81	0.76	0.35
Specificity	0.85 ± 0.01	0.79	0.93	0.72	0.90	0.82 ± 0.01	0.71	0.85	0.67	0.90
F1-Score	0.76 ± 0.03	0.87	0.89	0.73	0.56	0.72 ± 0.03	0.86	0.82	0.73	0.46
AUC	0.91 ± 0.02	0.93	0.95	0.78	0.43	0.86 ± 0.02	0.91	0.92	0.77	0.30
MCC	0.60 ± 0.01	0.73	0.81	0.45	0.76	0.57 ± 0.01	0.71	0.67	0.44	0.68

Evaluation of training dataset and three testing dataset using AUC (Area Under Curve), ACC (Accuracy), Precision, Sensitivity, Specificity, F1 score together with MCC (Matthews correlation coefficient).

The performance of our method was compared not only by internal cross validation but also by three autonomous external validation datasets. The threshold value of prediction scores identifying deleterious variants were varied to find their optimums, keeping the best sensitivity and specificity. Based on both true and false positives, as well as both true and false negatives, all other corresponding criteria were measured. For training dataset, tenfold cross validation was repeated ten times, as a result, we reported mean of all criteria with variance. For three-source algorithm the specificity and area under the Curve (AUC) resulted in 0.85 and 0.91, respectively, whereas for two-source algorithm, they were obtained in respective 0.82 and 0.86 (Table 2). Comparing results in three test datasets, we found that our three-source algorithm is superior over two-source algorithm. The inclusion of gene level data or gene disease association at three-source algorithm substantially influence the function prediction of variants. For the first three datasets, all criteria were increased except for sensitivity which decreased from 0.95 to 0.92 and 0.76 to 0.73 for testing dataset I and testing dataset III, respectively. This confirms that gene disease relations can improve prediction rather than variant level information.

### Evaluation of type 2 circularity in different prediction methods and our proposed method

When a classifier utilizes gene level information to mainly predict deleterious variants using known information in variants which are located in same genes, Type 2 circularity occurs. In such tools, there is a tendency to label all variants harbored by the same gene as well as same labels, either deleterious or neutral.

To investigate the extent to which our method influenced by circulatory error 2, we tested our method for other two different datasets; testing dataset III and VI. Variants encompassed at testing dataset III located on genes which harbor both neutral and deleterious variants named as “mix” gene. On the other hand, variants of testing dataset IV which are located in same genes are labeled as all deleterious or neutral. These genes are called as “pure” genes. As a result, the superior prediction results for testing dataset IV against testing dataset III would indicate which the classifier is overfitted and type 2 circularity is taken placed.

**Figure 3:**
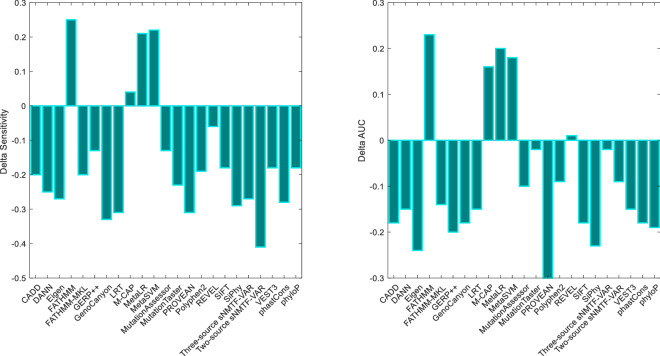
The variation of sensitivity (Right) and AUC (Left) of testing dataset IV in comparison with testing dataset III for different prediction tools and both algorithm of our sNMTF-VAR method. The tools which their bars are over the base line were affected by type 2 circularity error. *The figure is created using MATLAB version 7.0.1 (Math Software Co., Math Works, USA).*

As can see from Fig. 3, the sensitivity of testing data IV was considerably decreased for both two-source and three-source algorithms in comparison with testing dataset III. It is indicated that our algorithm is not suffered from circularity error 2. On the contrary, the specificities were significantly inflated from 0.67 to 0.90 and 0.72–0.90 for two-source and three-source algorithms, respectively (Table 2). In fact, our prediction method, labels most of variants at testing data IV as neutral so the accuracy does not drastically change and specificity is increased against testing dataset III. The explanation behind is that our method cannot identify deleterious variants in “pure” genes because it does not annotate all variants that located on pathogenic genes as deleterious. Using previous variant effect scores at relation matrix, Rvs, could cause our method not to predict variants only based on genes harbor variants.

As it is evident from Fig. 3, all methods except for FATHMM, MCAP, MetaSVM and MetaLR were not influenced by circulatory error 2 as a result of substantial increase in both AUC and sensitivity in testing data IV. For the first time, Grimm and coworkers proved this fact for FATHMM but they did not perform any experiment about recent ensemble methods like MCAP, MetaSVM and MetaLR, REVEL, FATHMM-MKL, DANN, GenoCanyon and Eigen. As FATHMM is one of the attribute of MCAP, MetaSVM and MetaLR which are composed of, the performance of these ensemble based methods improved for testing data IV. All obtained criteria was shown in Table S2 and S3for testing dataset III and testing dataset IV. Also, ROC curves were plotted regarding different prediction tools for testing dataset III at Figure S2. As the testing dataset IV is an artificial dataset which was constructed for evaluation of type 2 circulatory, we did not compare the AUCs of different methods.

### Superiority over existing methods

**Figure 4:**
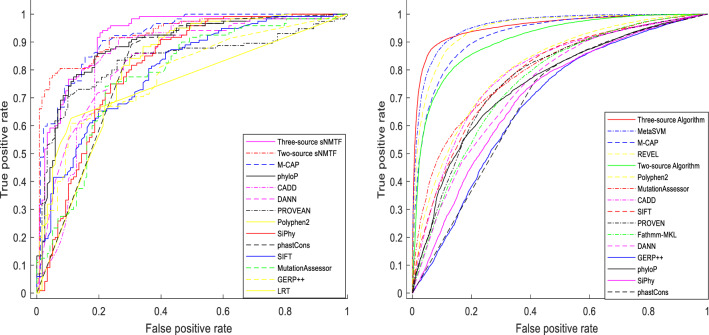
ROC curves of different methods at testing dataset I (right) and testing dataset II (left). *The figure is created using MATLAB version 7.0.1 (Math Software Co., Math Works, USA).*

Our two algorithms were advantageous to all their constituent variant effect prediction scores which used for variant-score relation matrix construction for testing data I (Table 3), testing data II and testing data III (Table S1, S2) in the case of all criteria. The best AUCs among nine constituent prediction methods are belong to Phylop (AUC = 0.89), Polyphen2 (AUC = 0.81) and PROVEAN (AUC = 0.71) for testing data I, testing data II and testing data III, respectively. While these values are 0.93, 0.95, and 0.78 for three-source, and 0.91, 0.92, and 0.77 for two-source algorithm. Of all our utilized prediction scores, similar with other research outputs, functional scores (SIFT, MutationAssessor, PROVEAN, PolyPhen-2 and LRT) have better prediction results than conservation scores (SiPhy, PhastCons, PhyloP, and GERP++). In addition of nine constituent variant effect prediction scores, we compared our method with other ensemble tools. ROC curve of all methods were plotted as Fig. 4 for Testing data I and Testing data II. Figure S2 compares the ROC curves of all methods for Testing Data III. For testing data I, the best ROC curves are belong to our method and REVEL simultaneously. Moreover, for Testing data II this is the case for three-source algorithm, MetaSVM and MetaLR, whereas REVEL and M-CAP are in the second place. Also, two-source algorithm is on the third. Furthermore, three–source algorithm has the highest accuracy, F1 score and MCC for three testing data and is superior to all methods including ensemble based ones. In overall, according to the three first sets of benchmark data, we found that our proposed predictor and REVEL methods had the maximum functions.

**Table 3:** Performance evaluation based on benchmark test data I: *ACC* accuracy, *MCC* Matthews correlation coefficient, and *AUC* area under curve.

Methods	ACC	Precision	Sensitivity	Specificity	F1	MCC	AUC
SIFT	0.72	0.70	0.77	0.67	0.74	0.44	0.78
Polyphen2	0.76	0.72	0.87	0.66	0.79	0.54	0.81
LRT	0.79	0.77	0.89	0.67	0.83	0.58	0.76
MutationAssessor	0.70	0.70	0.75	0.65	0.72	0.41	0.80
PROVEAN	0.77	0.76	0.80	0.74	0.78	0.55	0.83
MetaSVM	0.80	**0.89**	0.69	**0.91**	0.78	0.62	0.90
MetaLR	0.79	0.88	0.69	0.90	0.77	0.61	0.91
M-CAP	0.81	0.77	0.95	0.64	0.85	0.63	0.92
CADD	0.78	0.72	0.93	0.62	0.81	0.59	0.84
DANN	0.77	0.74	0.83	0.71	0.79	0.55	0.84
FATHMM-MKL	0.74	0.68	0.93	0.56	0.79	0.53	0.86
GERP++	0.65	0.60	0.92	0.39	0.73	0.37	0.78
phyloP	0.79	0.74	0.91	0.67	0.81	0.60	0.89
phastCons	0.79	0.74	0.89	0.69	0.81	0.60	0.81
SiPhy	0.75	0.73	0.79	0.71	0.76	0.50	0.81
REVEL	0.85	0.85	0.85	0.85	0.85	0.70	0.93
Two-source sNMTF-VAR	0.84	0.77	**0.95**	0.71	0.86	0.71	**0.91**
Three-source sNMTF-VAR	**0.86**	0.82	0.92	0.79	**0.87**	**0.73**	**0.93**

### Comparison of prediction results on variants located on pathogenic and nonpathogenic genes

**Figure 5:**
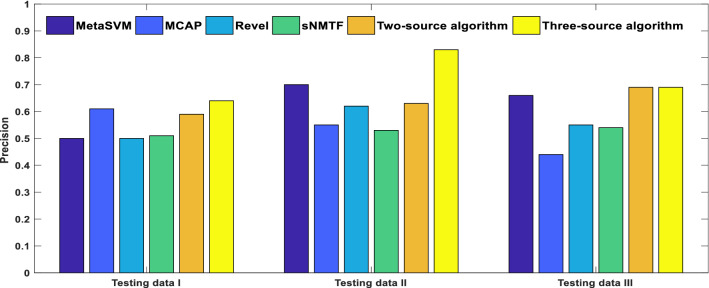
The bar charts represented the precision values of the six different deleteriousness prediction tools for the variants positioned on nonpathogenic genes over three testing datasets. *The figure is created using MATLAB version 7.0.1 (Math Software Co., Math Works, USA).*

As we did at our previous work^37^ in which we compared the prediction performance in two types of variant located on disease related genes and others, we categorized our datasets to two parts according to types of genes hourberd variants, and we measured our method and other deleterious variant prediction methods performance including our previous method, sNMTF, for both of nsSNP types. As clearly shown at Fig. 5, our three-source algorithm has higher precision for variants located on nonpathogenic genes over three datasets. It is indicated that for our predictor, in comparison with other ensemble based methods, we can rely on variants annotated as deleterious even though we do not have enough information about the genes located on. Tables S4, S5 and S6 illustrate the other criteria obtained from variants placed on nonpathogenic genes. Both versions of our method (Two-source & three-source) have higher performance in most of criteria over other methods.

## Discussion and conclusion

In this study, we presented a new integration scheme of different omics data types at varying levels to progress the variant effect prediction methods. Our proposed framework, with sharing relevant information of nsSNVs at PPI network, functional deleteriousness scores, gene disease associations and nsSNV data to distinguish between deleterious and neutral variants. We developed two algorithms of our classifier. Two-source algorithm utilized PPI network and functional scores assembling variant-variant and score-score network, respectively. Three-source algorithm, incorporated disease gene relations and disease similarities in the form of variant-score relation matrix together with disease-disease network. Two relation matrices, R_VS_ and R_VD_, simultaneously were factorized to low dimension space at train step. Consequently, by means of learned cluster indicator matrices of the scores and disease data, we obtained class indicator matrix of variants and predict the labels at testing step.

The prominent mark of our predictor is integration of gene-disease association data with large amount of information at variant level data including predicted deleteriousness effect of nsSNVs and network based features. It was considered in tools such as eXtasy^51^, snvforest^18^. The basis of these methods are rely on extraction of various type of features, variant level and gene-disease association features, then applying a classifier such as random forest, SVM or gradient boosting on these feature sets. Undoubtedly, the performance of these methods is dependent on proper feature extraction and selection. On the other hand, tools based on extracting features transforming all types of features to one common feature space cause to have loss of information. Contradictorily, to address these problems we regarded variant-variant, score- score and disease-disease networks in integration framework, and involve intrinsic structure of all networks instead of easy extraction of some limited features. It was proved that classification accuracy is strongly depends on the way that different types of data integrate especially when the data becomes very big and heterogeneous. As a result, integration strategies which are able to concurrently construct the predictive model and take into account the overall structure of all input data sources and their relationships, are preferred to the rest of integration strategies^52^. At our previous work, for the first time, we applied this strategy for variant effect prediction^37^. Despite of superior prediction of our sNMTF method, this method did not have reasonable performance for variants located on non-pathogenic genes. In addition, it used the functional scores indirectly via constructing variant-variant network. So, in this study we considered functional scores as relation matrix of variant-score to straightforwardly have effect on prediction results especially on variants located on genes which there is no sufficient disease related knowledge and cannot significantly help us to distinguish deleterious variants.

An innovation of our new method is the way that it uses to supervise the NMTF clustering methods. At sNMTF method we made a feature map on a low dimensional space using NMTF. Then as a supervised frame the variant labels alongside features imported to a random forest classifier. The advantage of recent algorithm is that we did not consider an additional classifier, instead we imported labels to objective function at training step, and by means of the learned factorized matrices we concluded deleteriousness effect of test variants. Consequently, the elimination of the classifier in this method decreases the complexity of model.

The other highlight of presented work is handling PPI network to build variant-variant network. For the first time, Leal and his colleagues made use of this idea to pritorize variants by aggregating PPI, variant damaging effect, genotype, phenotype and subjects’ ancestry^32^. In their variant-variant network, they connected all variants located in same genes but give more weights to connections of deleterious variants. In the case of variants stated at different genes, only deleterious ones were connected to each other. This configuration brings bias to variant effect prediction. Hence, we equally weighted all of connections between variants on same genes and did not give preference to damaging variants at different genes. These variants were weighted according to edge score connecting corresponding proteins to each other at PPI network. The previous studies expressed the importance of PPI network topology in variant effect prediction^26,29^ by extraction of proper features from PPI network. Through the way we constructed our variant-variant network we maintained the whole structure of PPI network at variant network including being located of the deleterious variants at hub proteins instead of extraction of some features of PPI network.

We did Extensive review on variety of test datasets to investigate two algorithms of our method, two-source and three-source algorithm, along with previous prediction tools. Our both algorithms have a superior performance to original constituent variant prediction scores for three independent test datasets. Overall, three-source algorithm has preference for almost all ensemble-based prediction methods. We regarded the performance of our predictor in the presence of variant and gene levels information. In the case of gene level data, it was demonstrated that the specificity value was notably increased at three-source algorithm against two-source one. This indicated that gene-disease association data has an impressive role to play in accurate identification of neutral variants from other ones. But incorporating gene information into predictor, it is important to take into account that it is not affected by circulatory error 2. By comparison of results for testing dataset III and IV, the quality of suffering from type 2 circularity was assessed. It was clear that MetaSVM, MetaLR and M-CAP were affected by this error while our both algorithms were not influenced. In the case of variant level data, it was proved that we can fall back more in deleterious variants located on nonpathogenic genes which were predicted via our method in comparison with other prediction methods including our previous method, sNMTF. In other words, in these kinds of variants which we do not have any information of gene-disease association, algorithm can well predict variants, because variant level data was incorporated to our network.

Since the obtained predicted results expressively varies in different testing dataset we investigated the characteristics of the all sets of data. By way of the same ratio of variants placed on disease related genes for all datasets, it cannot explain the difference among the obtained results. Also, for the two-source algorithm we cannot rely on it, because we did not use disease-gene association information on this algorithm. Notably, according to the Table 1, the ratio of variants placed on pure genes for dataset D-III is very fewer than the other datasets. Thus, it is reasonable to assume a correlation between the ratio of variants placed on the pure genes and the obtained performance in each dataset. However, considering the performances in dataset D-I and D-II reveals that we cannot confidently state this hypothesis. In other words, the ratio of variants placed on disease related genes for dataset D-III is much lower than other datasets, which can explain its worst performance for the three-source algorithm. On the other hand, according to the Table 2, the performance increases for the three-source algorithm compared to the two-source one in dataset D-III. But this increase for other two datasets is much bigger than the increase for dataset D-III. It is evident, because very low information was added to the network for the three-source algorithm (gene-disease information). This phenomenon was created because of self-adjustment of our recent model. Once we do not aware of the pathogenicity of harbored gene, the workflow can adjust itself to some extent by other types of data such as functional scores, topology of PPI network and disease-disease relation, and vice versa. In the case in which variant level data do not have a major contribution in classifying deleterious variants and neutral ones, workflow regards gene disease associations.

Finally, in order to reduce the convergence time of objective functions, multiplicative updating rules have the potential to be substituted by optimization algorithms such as projected gradients, coordinate descent and alternating least squares methods. Even we can replace or add more data sources to our proposed method for variant deleteriousness prediction. An alternative can be allele frequency to provide us with checking the rare variants. We can propose to verify such methods on other types of variants rather than nsSNPs.

## Data availability

The datasets generated during and analysed during the current study are available from the corresponding author on reasonable request.

## Supplementary Information

Supplementary Information.
