# Supplementary material for: Predicting deleterious missense genetic variants via integrative supervised nonnegative matrix tri-factorization
Source: Sci Rep. 2021 Dec 9;11:23747. doi: 10.1038/s41598-021-03230-x (PMC8660898; doi:10.1038/s41598-021-03230-x)
Supplement: Supplementary file 1 — Supplementary Information. [file 41598_2021_3230_MOESM1_ESM.pdf]

Supplementary Materials

for

Predicting deleterious missense genetic variants via integrative supervised nonnegative matrix tri-factorization

Asieh Amousoltani Arani[1,2], Mohammadreza Sehhati[3, 4,*], Mohammad Amin Tabatabaiefar[4,5]

[1] Department of Bioelectric and Biomedical Engineering, School of Advanced Technologies in Medicine, Isfahan University of Medical Sciences, Isfahan, Iran

[2] Student Research Committee, School of Advanced Technologies in Medicine, Isfahan University of Medical Sciences, Isfahan, Iran

[3] Department of Bioinformatics, School of Advanced Technologies in Medicine, Isfahan University of Medical Sciences, Isfahan, Iran

[4] GTaC Corp., Deputy of Research and Technology, Isfahan University of Medical Sciences, Isfahan, Iran

[5] Department of Genetics and Molecular Biology, School of Medicine, Isfahan University of Medical Sciences, Isfahan, Iran

Supplementary Tables & Figures

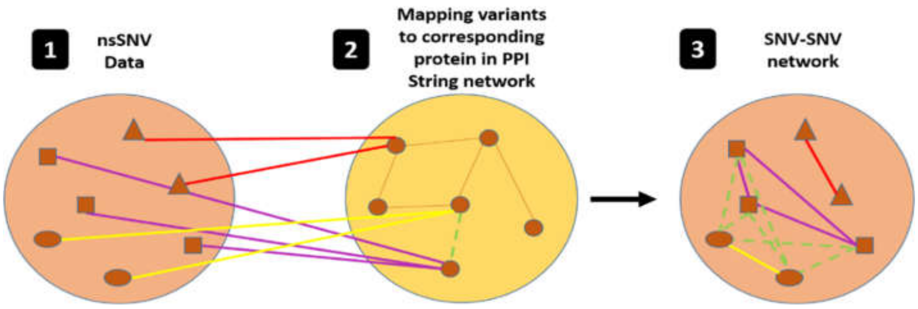

Figure S1: construction of variant-variant network via PPI network. nsSNVs which are illustrated at the same shape, are located on the same genes. For example, in this nsSNV dataset, we have three variants (shown with square) located on a gene. Each variant was mapped to corresponding protein on PPI network (violet lines). If there was a connection between pair of proteins, all their variants would connect to each other (dashed green lines between squares and circles). In addition, all variants placed at the same genes were connected to each other (not dashed lines at network number 3).

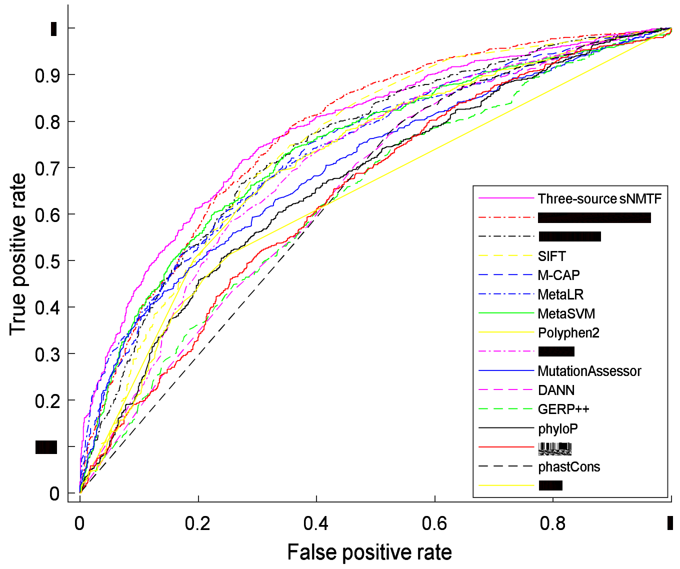

Figure S2: ROC curves of different predictor tools for testing dataset III.

Table S1: Performance evaluation based on benchmark testing data II. ACC = accuracy, MCC = Matthews correlation coefficient, and AUC = area under curve.

Methods	ACC	Precision	Sensitivity	Specificity	F1	MCC	AUC
**SIFT**	0.71	0.66	0.78	0.64	0.72	0.43	0.78
**Polyphen2**	0.72	0.67	0.83	0.74	0.47	0.46	0.81
**LRT**	0.70	0.64	0.77	0.63	0.70	0.40	0.70
**MutationAssessor**	0.69	0.67	0.77	0.66	0.71	0.36	0.80
**PROVEAN**	0.72	0.28	0.86	0.70	0.42	0.38	0.77
**MetaSVM**	0.89	0.90	0.86	0.91	0.87	0.76	**0.95**
**MetaLR**	0.89	0.90	0.87	0.90	0.87	0.77	**0.95**
**M-CAP**	0.83	0.81	**0.97**	0.50	**0.89**	0.59	0.92
**CADD**	0.70	0.63	0.87	0.54	0.73	0.43	0.79
**DANN**	0.68	0.63	0.77	0.60	0.70	0.37	0.74
**FATHMM-MKL**	0.65	0.61	0.86	0.44	0.71	0.37	0.76
**GERP++**	0.60	0.54	0.89	0.33	0.68	0.27	0.68
**phyloP**	0.66	0.61	0.80	0.53	0.69	0.35	0.75
**phastCons**	0.67	0.62	0.76	0.58	0.68	0.35	0.69
**SiPhy**	0.67	0.64	0.70	0.64	0.67	0.34	0.70
**REVEL**	0.87	0.84	0.90	0.85	0.87	0.75	0.94
**Two-source sNMTF-VAR**	0.83	0.83	0.81	0.85	0.82	0.67	0.92
**Three-source sNMTF-VAR**	**0.91**	**0.92**	0.87	**0.93**	**0.89**	**0.81**	**0.95**

Table S2: Performance evaluation based on benchmark testing data III. ACC = accuracy, MCC = Matthews correlation coefficient, and AUC = area under curve.

**Methods**	**ACC**	**Precision**	**Sensitivity**	**Specificity**	**F1**	**MCC**	**AUC**
**SIFT**	0.67	0.64	0.76	0.57	0.70	0.34	0.74
**Polyphen2**	0.64	0.60	0.85	0.43	0.70	0.31	0.71
**LRT**	0.64	0.61	0.78	0.50	0.69	0.3	0.63
**MutationAssessor**	0.64	0.63	0.70	0.58	0.66	0.28	0.70
**FATHMM**	0.64	0.60	0.67	0.61	0.68	0.30	0.69
**PROVEAN**	0.70	0.69	0.72	0.66	0.70	0.40	0.75
**MetaSVM**	0.68	0.70	0.65	0.71	0.67	0.37	0.74
**MetaLR**	0.67	0.68	0.68	0.67	0.68	0.35	0.73
**M-CAP**	0.56	0.56	**0.93**	0.25	0.70	0.25	0.74
**CADD**	0.64	0.60	0.84	0.44	0.70	0.31	0.70
**DANN**	0.62	0.60	0.77	0.47	0.67	0.26	0.65
**FATHMM -MKL**	0.60	0.56	0.90	0.30	0.70	0.26	0.67
**GERP++**	0.56	0.54	0.88	0.25	0.67	0.17	0.63
**phyloP**	0.60	0.58	0.76	0.44	0.66	0.21	0.66
**phastCons**	0.63	0.60	0.77	0.47	0.67	0.26	0.64
**SiPhy**	0.61	0.60	0.69	0.53	0.63	0.22	0.64
**REVEL**	0.71	0.69	0.78	0.65	**0.73**	0.43	**0.80**
**Two-source sNMTF-VAR**	0.72	0.70	0.76	0.67	0.73	0.44	0.77
**Three-source sNMTF-VAR**	**0.73**	**0.72**	0.73	**0.72**	**0.73**	**0.45**	0.78

Table S3: Performance evaluation based on benchmark data testing data IV. ACC = accuracy, MCC = Matthews correlation coefficient, and AUC = area under curve.

Methods	ACC	Precision	Sensitivity	Specificity	F1	MCC	AUC
**SIFT**	0.53	0.40	0.58	0.50	0.48	0.08	0.56
**Polyphen2**	0.55	0.43	0.66	0.50	0.52	0.15	0.62
**LRT**	0.47	0.36	0.47	0.48	0.40	0.04	0.48
**MutationAssessor**	0.55	0.42	0.57	0.55	0.49	0.11	0.60
**FATHMM**	0.87	0.77	0.92	0.84	0.84	0.74	0.92
**PROVEAN**	0.50	0.35	0.41	0.56	0.38	0.02	0.45
**MetaSVM**	0.85	0.75	0.87	0.84	0.81	0.70	0.92
**MetaLR**	0.85	0.75	0.89	0.83	0.81	0.70	0.93
**M-CAP**	0.69	0.56	0.97	0.50	0.71	0.50	0.90
**CADD**	0.48	0.37	0.64	0.39	0.47	0.03	0.52
**DANN**	0.50	0.37	0.52	0.50	0.43	0.02	0.50
**FATHMM -MKL**	0.43	0.36	0.70	0.30	0.47	-0.01	0.53
**GERP++**	0.41	0.35	0.75	0.22	0.48	-0.03	0.43
**phyloP**	0.43	0.34	0.58	0.35	0.43	-0.06	0.47
**phastCons**	0.46	0.33	0.49	0.44	0.40	-0.06	0.46
**SiPhy**	0.45	0.31	0.40	0.49	0.35	-0.10	0.41
**REVEL**	0.72	0.60	0.72	0.73	0.66	0.44	0.81
**Two-source method**	0.70	0.66	0.35	0.90	0.46	0.30	0.68
**Three-source method**	0.74	0.76	0.46	0.90	0.56	0.43	0.76

Table S4: Evaluation of the six different deleteriousness prediction tools including our proposed methods and sNMTF for variants located on nonpathogenic genes over testing data I.

Methods	ACC	Precision	Sensitivity	Specificity	MCC
**MetaSVM**	0.73	0.50	0.18	**0.93**	0.17
**M-CAP**	0.82	0.61	**0.95**	0.75	**0.67**
**Revel**	0.73	0.50	0.36	0.87	0.26
**sNMTF**	0.75	0.51	0.58	0.81	0.37
**Two-source sNMTF-VAR**	**0.80**	0.59	0.90	0.77	0.60
**Three-source sNMTF-VAR**	0.78	**0.64**	0.72	0.83	0.53

Table S5: comparison of our proposed method performance and other prediction methods including sNMTF for variants located on nonpathogenic genes of Testing data II.

Methods	ACC	Precision	Sensitivity	Specificity	MCC
**MetaSVM**	0.95	0.70	0.88	0.90	0.77
**M-CAP**	0.75	0.55	**0.98**	0.65	0.59
**Revel**	0.92	0.62	0.92	0.92	0.72
**sNMTF**	0.80	0.53	0.62	0.85	0.43
**Two-source sNMTF-VAR**	0.92	0.63	0.91	0.93	0.72
**Three-source sNMTF-VAR**	**0.96**	**0.83**	0.89	**0.97**	**0.95**

Table S6: comparison of our proposed method performance and other prediction methods including sNMTF for variants located on nonpathogenic genes of Testing data III.

Methods	ACC	Precision	Sensitivity	Specificity	MCC
**MetaSVM**	0.74	0.66	0.72	**0.75**	0.47
**M-CAP**	0.50	0.44	**0.91**	0.22	0.18
**Revel**	0.66	0.55	0.86	0.52	0.39
**sNMTF**	0.72	0.54	0.66	0.68	0.34
**Two-source sNMTF-VAR**	0.78	0.69	0.86	0.73	**0.59**
**Three-source sNMTF-VAR**	**0.79**	**0.69**	0.86	0.73	**0.58**

Methods

Minimizing objective functions

To solve our optimization function of equation 5, we broke it into four subproblems: derivation of U in regarding to the V, S, and G_Y are fixed; derivation of V when the S, U, and G_Y are constant; derivation of S once the V , U, and G_Y do not change; derivation of G_Y with regard to the V , U, and S are consistent. These four subproblems updates all values iteratively until predefined termination criterion is achieved. Multiplicative updating rules are using deriving such problems which are non-convex[1]. The process is started with initialization of matrix factors, V , S, U and G_Y matrices using random acol strategy[2]. As a result, the updated rule for four matrices are given below:

$$U \leftarrow (V^T V)^{-1}[V^T US + V^T YG_Y][S^T S + G_Y^T \ G_Y]^{-1} \tag{S1}$$

$$V \longleftarrow V\sqrt{\frac{(R_{VS}SU^T)^+ + [V(US^{\ T}SU^T)^-] + \gamma_1(L_V^- V) + (Y^{tr}G_Y S)^+ + V(UG_Y^T G_Y U^T)^-}{(R_{VS}SU^T)^- + [V(US^{\ T}SU^T)^+]_{(m,n)} + \gamma_1(L_V^+ V) + (Y^{tr}G_Y S)^- + V(UG_Y^T G_Y U^T)^+}} \tag{S2}$$

$$S \longleftarrow S\sqrt{\frac{(R_{VS}^T VU)^+ + [S(UV^{\ T}VU^T)^-] + \gamma_2(L_S^- S)}{(R_{VS}^T VU)^- + [S(UV^{\ T}VU^T)^+] + \gamma_2(L_S^+ S)}} \tag{S3}$$

$$G_Y = \sqrt{\frac{(Y^T VU)^+ + G_Y(U^T V^T VU)^-}{(Y^T VU)^- + G_Y(U^T V^T VU)^+}} \tag{S4}$$

While, X^+ and F^- are nonnegative matrix as below:

$$X^+ = \begin{cases} F & if\, F \geq 0 \\ 0 & \text{else} \end{cases} \qquad X^- = \begin{cases} F & if\, F \leq 0 \\ 0 & \text{else} \end{cases} \tag{S5}$$

In a similar manner, we solve the objective function number 6 and matrices given as:

$$V^{ts} \longleftarrow V^{ts}\sqrt{\frac{(R_{VS}^{ts}SU_{ts}^T)^+ + [V^{ts}(U^{ts}S^{\ T}SU_{ts}^T)^-] + \gamma_1(L_V^- V^{ts})}{(R_{VS}^{ts}SU_{ts}^T)^- + [V^{ts}(U^{ts}S^{\ T}SU_{ts}^T)^+] + \gamma_1(L_V^+ V^{ts})}} \tag{S6}$$

$$U^{ts} \leftarrow (V^{(ts)T}V^{ts})^{-1}[V^{(ts)T}U^{ts}S](S^T S)^{-1} \tag{S7}$$

The deriative matrices for three-source algorithm, solving equation 8 are such as following:

$$U_1 \leftarrow (V^T V)^{-1}[V^T U_1 S + V^T Y G_Y][S^T S + G_Y^T\ G_Y]^{-1} \quad \text{(S8)}$$

$$U_2 \leftarrow (V^T V)^{-1}[V^T U_2 D][D^T D]^{-1} \quad \text{(S9)}$$

$$V \longleftarrow V\sqrt{\frac{(R_{VS}SU_1^T)^+ + [V(U_1S^TSU_1^T)^-] + (R_{VD}DU_2^T)^+ + V(U_2D^TDU_2^T)^- + \gamma_1(L_V^-V) + (YG_YU_1)^+ + V(U_1G_Y^TG_YU_1^T)^-}{(R_{VS}SU_1^T)^- + [V(U_1S^TSU_1^T)^+] + (R_{VD}DU_2^T)^- + V(U_2D^TDU_2^T)^+ + \gamma_1(L_V^+V) + (YG_YU_1)^- + V(U_1G_Y^TG_YU_1^T)^+}} \quad \text{(S10)}$$

$$S \longleftarrow S\sqrt{\frac{(R_{VS}^TVU_1)^+ + [S(U_1V^TVU_1^T)^-] + \gamma_2(L_S^-S)}{(R_{VS}^TVU_1)^- + [S(U_1V^TVU_1^T)^+] + \gamma_2(L_S^+S)}} \quad \text{(S11)}$$

$$D \longleftarrow D\sqrt{\frac{(R_{VD}^TVU_2)^+ + [D(U_2V^TVU_2^T)^-] + \gamma_3(L_D^-D)}{(R_{VD}^TVU_2)^- + [D(U_2V^TVU_2^T)^+] + \gamma_3(L_D^+D)}} \quad \text{(S12)}$$

$$G_Y = G_Y\sqrt{\frac{(Y^TVU_1)^+ + G_Y(U_1^TV^TVU_1)^-}{(Y^TVU_1)^- + G_Y(U_1^TV^TVU_1)^+}} \quad \text{(S13)}$$

So the derived matrices of $V^{ts}, U_1^{ts}, U_2^{ts}$ at the testing phase (solving equation 9) are as:

$$V^{ts} \longleftarrow V^{ts}\sqrt{\frac{(R_{VS}SU_1^T)^+ + [V^{ts}(U_1S^TSU_1^T)^-] + (R_{VD}DU_2^T)^+ + V^{ts}(U_2D^TDU_2^T)^- + \gamma_1(L_V^-V^{ts})}{(R_{VS}SU_1^T)^- + [V^{ts}(U_1S^TSU_1^T)^+] + (R_{VD}DU_2^T)^- + V^{ts}(U_2D^TDU_2^T)^+ + \gamma_1(L_V^+V^{ts})}} \quad \text{(S14)}$$

$$U_1^{ts} \leftarrow (V^{(ts)T}V^{ts})^{-1}[V_{ts}^T U_1^{ts} S][S^T S]^{-1} \quad \text{(S15)}$$

$$U_2^{ts} \leftarrow (V^{(ts)T}V^{ts})^{-1}[V^{(ts)T} U_2^{ts} S][S^T S]^{-1} \quad \text{(S16)}$$

Parameter selection

We needed three factorization rank for both algorithms, k_V, k_S and k_D that indicate the number of cluster of variants, scores, and diseases. To infer rank parameters, we applied the strategy to stabilize the classification results in each run. As a result, we estimated the stability by dispersion coefficient of consensus matrix (the mean of connectivity matrix) while ran the both algorithms many times[3].

There are two different groups of deleterious function prediction score at both algorithms. One of them is sequence conservation scores which determine the deleteriousness of a variant by identifying conserved nucleotide position across diverse species. Other group measures deleterious functional change of the protein by aligning multiple amino acid sequences. Furthermore, we choose the score rank, k_S=2 to classify two conservation and functional scores. For two other ranks, variant and disease, we performed a predefined interval of ($1 \le k_V \le 250$, $1 \le k_D \le 250$), computed the ranks which on the dispersion coefficient begins to fall. Thus we set the ranks as k_V=200 and k_D=100.

Penalization parameters

To guarantee that all objective functions were converged for some iterations, we adjusted the penalization between value of zero and the proportion of main factorization and regularization terms[4]. For two-source algorithm, the range of parameters are defined as:

$$\gamma_1 \in \left[0, \frac{\left\| R_{VS} - VUS^T \right\|_F^2 + \left\| Y - VUG_Y^T \right\|_F^2}{tr(VL_V V^T)} \right]$$

$$\gamma_2 \in \left[0, \frac{\left\| R_{VS} - VUS^T \right\|_F^2 + \left\| Y - VUG_Y^T \right\|_F^2}{tr(SL_S S^T)} \right]$$

For three-source algorithm, these ranges were changed to:

$$\gamma_1 \in \left[0, \frac{\left\| R_{VS} - VU_1 S^T \right\|_F^2 + \left\| R_{VD} - VU_2 D^T \right\|_F^2 + \left\| Y - VU_1 G_Y^T \right\|_F^2}{tr(VL_V V^T)} \right]$$

$$\gamma_2 \in \left[0, \frac{\left\| R_{VS} - VU_1 S^T \right\|_F^2 + \left\| R_{VD} - VU_2 D^T \right\|_F^2 + \left\| Y - VU_1 G_Y^T \right\|_F^2}{tr(SL_S S^T)} \right]$$

$$\gamma_3 \in \left[0, \frac{\left\| R_{VS} - VU_1 S^T \right\|_F^2 + \left\| R_{VD} - VU_2 D^T \right\|_F^2 + \left\| Y - VU_1 G_Y^T \right\|_F^2}{tr(DL_D D^T)} \right]$$

Consequently, we performed a grid search for $\gamma = \{0.0001\gamma_{\max}, 0.001\gamma_{\max}, 0.01\,\gamma_{\max}, 0.1\,\gamma_{\max}, \gamma_{\max}\}$ for all three regularization parameters. As a result, we set them in respective values of 10^{-4} and 0.1 for Gamma 1 and Gamma 2, for two-source algorithm, and 10^{-4}, 0.1, and 5 for Gamma 1, Gamma 2, and Gamma 3 for three-source algorithm, respectively.

References

1. Wang, F., Li, T. & Zhang, C. Semi-supervised clustering via matrix factorization. Soc. Ind. Appl. Math. - 8th SIAM Int. Conf. Data Min. 2008, Proc. Appl. Math. 130 **1**, 1–12 (2008).

2. Langville, A. N., Meyer, C. D., Albright, R., Cox, J. & Duling, D. Algorithms , Initializations , and Convergence for the Nonnegative Matrix Factorization. Arxiv (2014). doi:https://arxiv.org/abs/1407.7299

3. Kim, H. & Park, H. Gene expression Sparse non-negative matrix factorizations via alternating non-negativity-constrained least squares for microarray data analysis. Bioinformatics **23**, 1495–1502 (2007).

4. Leal, L. G. et al. Identification of disease-associated loci using machine learning for genotype and network data integration. Bioinformatics **35**, 5182–5190 (2019).
